# A Putative Blood-Based Biomarker for Autism Spectrum Disorder-Associated Ileocolitis

**DOI:** 10.1038/srep35820

**Published:** 2016-10-21

**Authors:** Stephen J. Walker, Daniel P. Beavers, John Fortunato, Arthur Krigsman

**Affiliations:** 1 Wake Forest Institute for Regenerative Medicine, Wake Forest University Health Sciences, Winston Salem, NC, USA; 2 Department of Biostatistical Sciences, Public Health Sciences, Wake Forest University Health Sciences, Winston Salem, NC, USA; 3 Pediatric Gastroenterology, Hepatology, and Nutrition, Ann & Robert H. Lurie Children’s Hospital of Chicago, Chicago, IL, USA; 4 Pediatric Gastroenterology Resources, 148 Beach 9th Street, Suite 2B, Far Rockaway, NY, USA.

## Abstract

Gastrointestinal symptoms are common in children with autism spectrum disorder (ASD). A significant proportion of children with ASD and gastrointestinal symptoms have histologic evidence of ileocolitis (inflammation of the terminal ileum and/or colon). We previously reported the molecular characterization of gastrointestinal biopsy tissue from ASD children with ileocolitis (ASD^IC+^) compared to anatomically similar inflamed tissue from typically developing children with inflammatory bowel disease (IBD; i.e. Crohn’s disease or ulcerative colitis) and typically developing children with gastrointestinal symptoms but no evidence of gastrointestinal mucosal inflammation (TD^IC−^). ASD^IC+^ children had a gene expression profile that, while primarily overlapping with known IBD, had distinctive differences. The present study confirms these findings and replicates this molecular characterization in a second cohort of cases (ASD^IC+^) and controls (TD^IC−^). In these two separate case/control mucosal-based cohorts, we have demonstrated overlap of 59 differentially expressed transcripts (DETs) unique to inflamed ileocolonic tissue from symptomatic ASD^IC+^ children. We now report that 9 of these 59 transcripts are also differentially expressed in the peripheral blood of the second cohort of ASD^IC+^ children. This set of transcripts represents a putative blood-based biomarker for ASD-associated ileocolonic inflammation.

It is established that gastrointestinal symptoms occur with greater frequency in children with ASD as compared to typically developing children, though the prevalence estimates of these symptoms vary depending on the methodologies employed^1^,^2^,^3^,^4^,^5^,^6^,^7^,^8^,^9^,^10^. Symptoms most often reported are constipation, diarrhea, abdominal pain and distention, and food intolerances^1^,^4^,^5^,^9^. Potential causes for these symptoms have included gastroesophageal reflux^10^, eosinophilic esophagitis^11^, food allergies^12^, and inflammatory bowel disease (IBD). IBD (Crohn’s disease and ulcerative colitis) was found to be 1.3–2.4 times more prevalent in children with ASD than in typically-developing children^13^.

Gastrointestinal symptoms in children with ASD have also been attributed to a unique variant of IBD seen only in children with ASD^9^,^14^. Distinguishing cellular, immunohistochemical, and molecular features of this ASD-associated IBD have been described in the literature from diagnostic endoscopies of the stomach^15^, small intestine^16^,^17^,^18^,^19^, and colon^17^,^19^,^20^. ASD-associated ileocolitis occurs in up to 70% of children undergoing diagnostic ileocolonoscopy^9^ –a much higher prevalence than the already increased incidence of “classic” IBD in these children^13^. The clinical significance of identification and treatment of ASD-associated ileocolitis and IBD extends beyond resolution of chronic gastrointestinal symptoms, especially given the association of these symptoms with extreme behavioral features of ASD^2^,^3^,^21^,^22^,^23^,^24^. Thus, treatment of symptomatic gastrointestinal disease in children with ASD may improve those symptoms and behavioral symptoms as well.

Historically, gastrointestinal symptoms in children with ASD have often either gone unrecognized^4^ or been treated empirically. Empiric treatment of a chronic condition such an IBD typically affords only transient improvement, if any. However, there are significant practical difficulties inherent in performing diagnostic endoscopy in children with ASD. Therefore, a blood biomarker that could reliably distinguish which symptomatic children with ASD are most likely to have IBD, and thus be most likely to benefit from diagnostic endoscopy, would be of enormous clinical value.

We have previously reported the molecular profile for histological ASD-associated ileocolitis that confirms, at the level of gene expression, the presence of ASD-associated IBD and discriminates it from Crohn’s disease and ulcerative colitis^19^. In the present study, we have extended those initial findings in an additional case/control cohort. We now report a gene expression profile in peripheral blood that may reflect the presence of ASD-associated ileocolitis and provide a putative surrogate biomarker that, upon validation, would be of significant clinical relevance.

## Results

### Demographic Characteristics of Cases and Controls

#### ASD^IC^^+^ samples

We collected samples from 21 children with a diagnosis of ASD (Table 1), chronic unexplained gastrointestinal symptoms (Table 2), and histologic inflammation of either the terminal ileum (N = 9), the colon (N = 9), or both (N = 3). For each individual, ileal and/or colonic biopsy specimens and a whole blood sample were processed.

#### Non-ASD [TD^IC−^] Controls

We collected samples from 24 typically-developing children without ASD who had gastrointestinal symptoms (Table 2), but no identifiable histologic inflammation on any biopsies in either the ileum or colon (TD^IC−^) (Table 1). The control children, on average, were older than the ASD children (12.6 years old versus 8.1 years old; Table 1). For each control individual, a terminal ileal or colonic biopsy specimen and a whole blood sample was processed.

### Principal Component Analysis (PCA)

**Figure 1:**
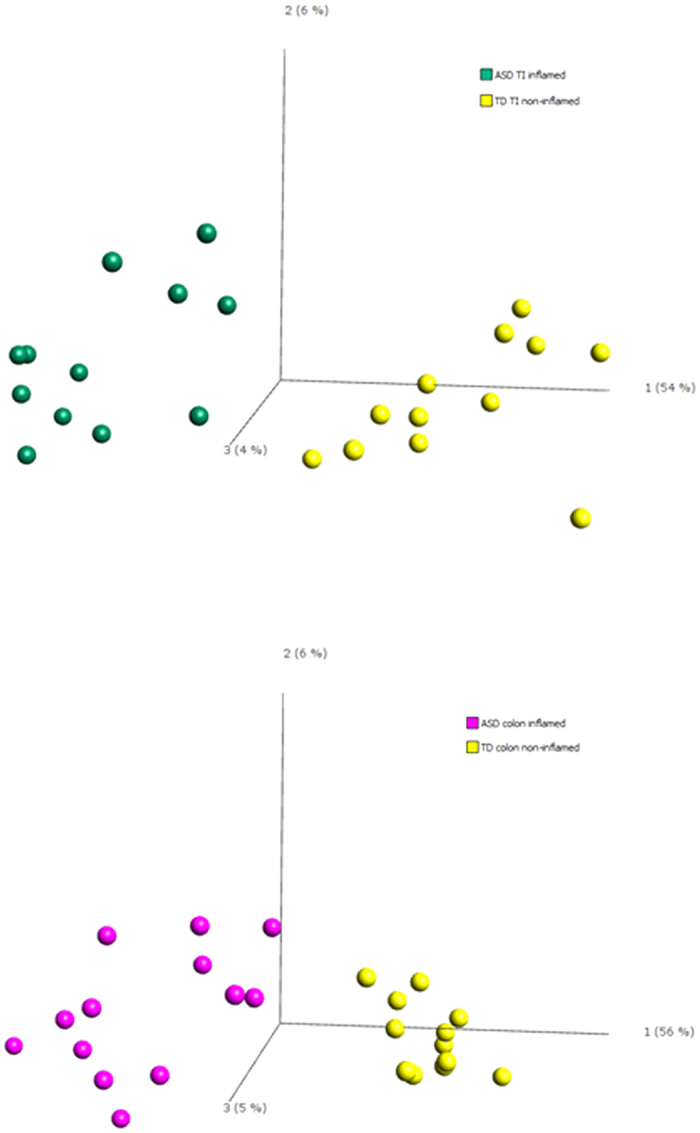
Principal component analysis. Gene expression profiles of inflamed terminal ileum tissue from ASD patients, compared to non-inflamed tissue from controls (top panel) and corresponding profiles in colonic tissue (bottom panel); p = 0.001. Teal = ASD-TI inflamed; Yellow = TD-TI non-inflamed; Violet = ASD colon inflamed; Yellow = TD colon non-inflamed.

Using principal component analysis, we compared whole genome gene expression profiles of inflamed ASD GI mucosal tissue (ASD^IC+^) to non-inflamed TD mucosal tissue (TD^IC−^) in biopsies from both the terminal ileum and colon. Consistent with what we reported in the original cohort^19^, mucosal-derived gene expression profiles, regardless of anatomic location, differed significantly between the inflamed and non-inflamed biopsy samples (Fig. 1).

#### Mucosal gene expression profiles

Control individuals cluster together in one area of the ileal PCA plot based on their gene expression profiles. The ASD^IC+^ individuals also cluster together, but in an area of the plot separate from controls (Fig. 1–top panel). This pattern was also seen in the colonic specimens, with the ASD^IC+^ individuals showing a separate and much broader distribution compared to controls (Fig. 1–bottom panel). In each case, the majority of separation between the groups (primarily due to inflammation status) is visualized in the first principal component (PC1).

#### Blood gene expression profiles

**Figure 2:**
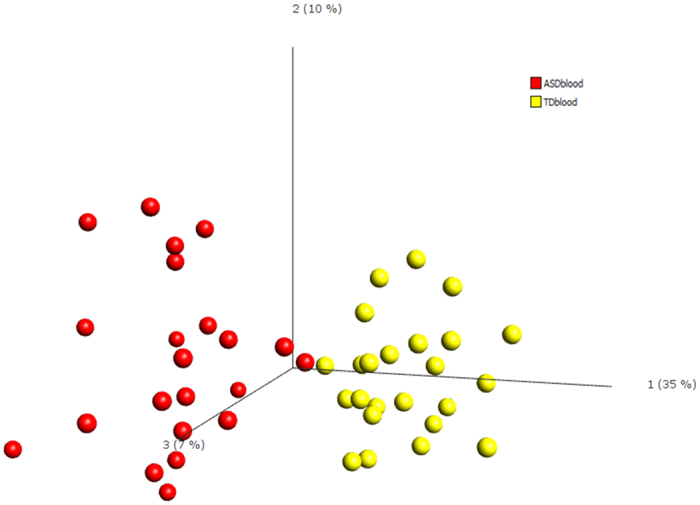
Principal component analysis. A comparison of gene expression profiles in peripheral blood from ASD patients with inflamed ileocolonic tissue compared to peripheral blood gene expression from TD controls without ileocolonic inflammation; p = 0.001. Red = ASD blood; Yellow = TD blood.

The second part of the current study was designed to identify transcripts that are differentially-expressed (DETs) in peripheral blood of ASD^IC+^ patients versus TD^IC−^ controls. Blood was obtained from the same patients, and at the same time, as their respective mucosal tissue samples and, similar to the findings in ilecolonic mucosal tissue sample comparisons, cases and controls are clearly separated based on their gene expression profiles (Fig. 2).

### Case:Control Comparisons

#### Overlapping differential gene expression GI biopsy tissue and peripheral blood

**Figure 3:**
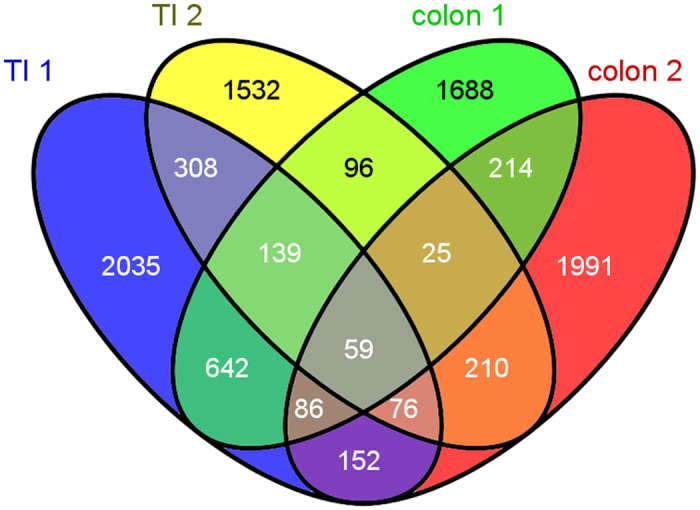
Differentially-expressed transcripts in two independent studies. Whole genome gene expression was measured in inflamed ileocolonic tissue (either terminal ileum or colon) from ASD patients with ileocolitis and compared to the corresponding non-inflamed tissue from non-ASD controls in two separate studies. The overlap in expression profiles is shown here. (TI 1 = terminal ileum data from the first study (25); TI 2 = terminal ileum data from the second study; colon 1 = colon data from the first study; colon 2 = colon data from the second study).

**Figure 4:**
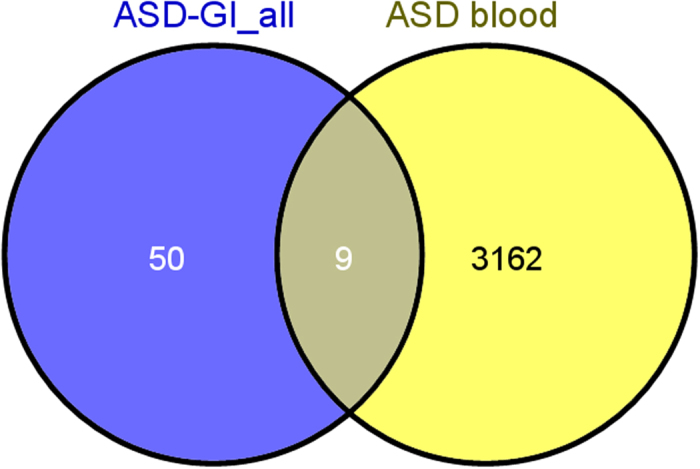
Overlapping gene expression. Genes that were uniquely differentially-expressed in inflamed gastrointestinal (GI) tissue from ASD patients in two separate studies, and the corresponding differential gene expression in blood from cases and controls (measured in the second study only) were compared to identify those DETs that occur in both tissues.

When we compared differential gene expression profiles in ileocolonic biopsy specimens from our original study^19^ and the current study, 59 overlapping DETs, common in both tissues–both studies, were identified (Fig. 3; Supplementary Table S1). The analysis of blood gene expression in ASD^IC+^ samples versus controls in the current study resulted in 3,171 differentially expressed transcripts (Supplementary Table S2). Comparison between the 59 mucosal-based DETs (both studies; Fig. 3 and Supplementary Tables S3–S6) with the blood-based DETs (from this study only) revealed 9 DETs in both blood and inflamed mucosa in *all* ASD^IC+^ cases (Fig. 4; Table 3). Four of these transcripts were up-regulated in both ileum and colon in all samples from both studies, and four were down-regulated. One DET, TNFRSF12A, is up-regulated in colon, but down-regulated in terminal ileum (Table 3). In the peripheral blood, the direction of differential expression for these DETs matched that in the mucosal tissue in five of nine instances (Table 3).

#### TaqMan validation of differential gene expression in peripheral blood

PCR validation of representative DETs in our earlier study showed significant agreement with microarray-based findings^19^. For the present study we compared the expression levels of five transcripts (CYP2S1 [1.38 fold ↓], TNFRSF12A [1.1 fold ↓], IL1RN [1.39 fold ↓], TNFAIP3 [2.39 ↑] and SIGLECP3 [1.35 ↓]) by PCR and found that in all cases the differential expression profile matched the microarray result in terms of direction of change (i.e. up- or down-regulated; Table 3).

### Receiver Operating Characteristic (ROC) Curve Analysis

**Figure 5:**
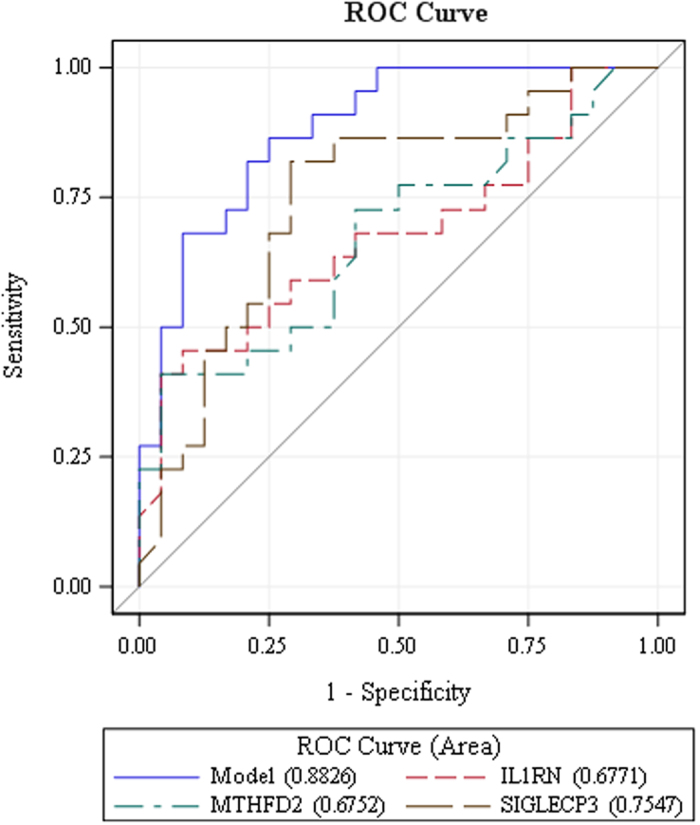
ROC analysis-blood. The variable selection procedure identified a linear combination of three transcripts that together yielded an AUC of 0.883.

To evaluate the predictive capability of the blood-based biomarkers, we performed an exploratory ROC analysis of the 9 transcripts differentially expressed in both blood and gastrointestinal tissue both univariately and in multivariable linear combinations. Areas under the curve (AUC) ranged from 0.627 to 0.755 among the individual differentially expressed blood-based transcripts (Supplementary Figure S1). A candidate composite biomarker was created using stepwise variable selection to identify a linear combination of transcripts that maximizes the AUC of a ROC curve, including biomarkers significant at a 0.05 level. The variable selection procedure identified a linear combination of three transcripts (MTHFD2, IL1RN and SIGLECP3) that together yielded an AUC of 0.883 (Fig. 5).

## Discussion

Here we describe a putative blood-based biomarker that may reflect the presence of ASD-associated ileocolitis. Differential gene expression findings in mucosal biopsies from our most recent cohort show significant overlap with, and provide validation of, results from our initial cohort. To our knowledge, this study represents the first effort to use such an approach in ASD children with gastrointestinal inflammation. In the earlier report, we described bowel tissue-derived DETs in children with ASD and gastrointestinal symptoms that were not present in typically developing children with Crohn’s disease or ulcerative colitis, or in typically-developing children without evidence of intestinal inflammation. Fifty-nine DETs were consistently seen in ileal and colon tissue biopsies from the two ASD^IC+^ cohorts and two TD^IC−^ control groups. Nine of these DETs were also differentially expressed in blood in our most recent cohort. Moreover, most of the nine transcripts identified in both ileocolonic biopsy tissue and blood from ASD^IC+^ children encode proteins associated with biologic processes known to be affected in children with ASD, suggesting the putative peripheral marker could provide a proxy for gastrointestinal inflammation and also provide functional insights.

Currently, a formal ASD diagnosis is based upon meeting DSMV criteria, often later than is desirable for commencing time-sensitive, maximally effective interventions. Thus, the search for diagnostic biomarkers capable of identifying at-risk children as early as possible has become a priority^25^,^26^,^27^. Numerous efforts are underway to identify diagnostically-relevant biomolecules (e.g. microRNAs^28^, mitochondrial DNA^29^, cytokines^30^, mRNAs^31^,^32^,^33^,^34^,^35^,^36^,^37^,^38^) in the peripheral blood of at-risk children. Given the broad heterogeneity that is the hallmark of ASD, coupled with the understanding that earlier diagnosis and treatment provides the greatest chance for the most positive outcomes, a blood-based test for early diagnosis of autism (and/or ASD subtypes) would have tremendous clinical value. Undoubtedly, at least initially, the most promising validated blood-based biomarkers will be derived from ASD subtypes. Thus, our current investigation is focused on the ASD subtype with comorbid gastrointestinal disorders.

Efforts to identify biomarkers for IBD are numerous. Diagnostic uncertainty resultant from clinical overlap between the two recognized types of IBD-Crohn’s disease and ulcerative colitis-have inspired many attempts to delineate IBD subtypes through comparisons of gene expression profiles in either mucosal biopsy tissue [e.g. refs 39, 40, 41, 42] or peripheral blood [e.g. refs 43, 44, 45, 46, 47]. As with ASD, arriving at an appropriate (and definitive) IBD diagnosis has important implications for early and successful therapeutic intervention and disease management. Because ileocolitis in ASD children shares many–but not all–clinical and molecular similarities with IBD, our original mucosal-based gene expression study in ASD^IC+^ children^19^ was modeled after the study by von Stein^41^ that reported a biomarker, consisting of seven transcripts, which could be used to distinguish Crohn’s disease from ulcerative colitis.

The rationale for seeking disease markers in peripheral blood begins with asking “To what extent does expression in white blood cells reflect expression in other organ systems”^48^. In an attempt to answer this question, several groups have focused on the overlap of gene expression in blood and brain tissues from diseased and control individuals^49^,^50^,^51^,^52^. In a recent review of eight brain/blood gene expression studies, 35% to 80% of known transcripts were found in both tissues; estimates of correlated (cross-tissue) expression levels ranged from 0.25–0.64, with the higher correlation found, not surprisingly, among specific subsets of genes^53^. A study of individuals with schizophrenia that reported gene expression levels in cadaveric brain tissue and peripheral blood from living patients found (and validated) a compelling biomarker candidate gene, *SELENBP1*^50^. A major limitation of these studies has been the quantity and quality of human banked tissue available for study^54^. A second important confound is that the brain gene expression data (often from brain bank tissues) and the blood gene expression data (often from living donors) typically do not come from the same individuals. By contrast, our study allowed us to evaluate both the affected organ tissue and blood, taken at the same time and from the same (living) individuals, to identify a clinically-relevant disease biomarker.

Our strategy for identifying a clinically-relevant peripheral biomarker for ASD^IC+^ is based on the premise that, whenever possible, biomarker discovery should begin in tissue that demonstrates known (and unique) disease-associated pathology to first identify a disease-specific signature^19^, followed by analysis of peripheral blood. Overlap of differential expression of specific transcripts within both sets of tissues provides additional confidence that the peripheral biomarker has validity and clinical relevance^50^.

Blood-based biomarkers have been reported for obstructive coronary artery disease^55^, Huntington’s disease^56^,^57^,^58^, multiple sclerosis^59^, epilepsy and new-onset idiopathic pediatric epilepsy^60^,^61^, and recent-onset juvenile idiopathic arthritis^62^, among others. Very few studies have used gene expression in the target “disease” tissue, correlated with peripheral blood gene expression in the same individuals, obtained simultaneously, to identify a blood-based biomarker.

Examination of terminal ileum and colonic specimens in this cohort was important, since ASD-associated ileocolitis has been observed in both the small bowel and colon. Evaluation of gene expression in both anatomic locations allowed us to identify common DETs and create an initial data set excluding transcripts whose differential expression may reflect not disease, but different tissue sites.

Most transcripts that comprise the putative blood-based biomarker have functions relevant in either ASD, inflammation, or both. For example *IL1RN* (interleukin 1 receptor antagonist), a potent anti-inflammatory molecule that inhibits the activities of IL1α and IL1β and modulates IL1-related immune and inflammatory responses, is upregulated in inflamed gastrointestinal tissues and down-regulated in the peripheral blood. Elevated levels of *IL1RN* in inflamed gastrointestinal mucosa makes biological sense in the context of the body’s attempt to modulate the damaging effects of the pro-inflammatory interleukin-1 in the gut and the well-established role of IL-1 in gastrointestinal inflammatory disorders^63^. Lower circulating levels of *IL1RN* may be a peripheral signal of the active inflammatory response in the gastrointestinal tract.

IL-1 also plays a major role in neuroinflammation^64^ and contributes to neuroinflammatory-associated breakdown of the blood-brain barrier^65^. The IL-1 family of cytokines is one of many pro-inflammatory cytokines present in excess in children with autism^66^,^67^,^68^, and both neuroinflammation and deficits in blood brain-barrier function have been implicated in the pathogenesis of ASD-related brain dysfunction^69^.

An important cytokine receptor transcript in this putative biomarker, *TNFRSF12A* (tumor necrosis factor receptor superfamily 12A), is over-expressed in inflamed colonic tissue and down-regulated in the terminal ileum and peripheral blood of ASD^IC+^ cases. This receptor (also called Fn14) binds the tumor necrosis factor superfamily member TWEAK (TNF-like weak inducer of apoptosis), a pro-inflammatory cytokine implicated in tissue regeneration and wound repair^70^. The binding of TWEAK to its receptor activates several signaling cascades, including the NF-κB pathway, and sustained Fn14 signaling has been implicated in the pathogenesis of chronic IBD. Moreover, TWEAK-independent Fn14 signaling may occur in instances where Fn14 levels are highly elevated^70^. Elevated Fn14 expression correlates highly with elevated *MET* (a hepatocyte growth factor receptor that encodes tyrosine kinase activity) in a form of metastatic cancer, and Fn14 depletion is sufficient to inhibit MET-driven tumor cell migration and invasion *in vitro*^71^. The human *MET* gene is a well-established risk factor for ASD that functions in both brain development and gastrointestinal repair, and confers a distinct risk in families with co-occurring autism and gastrointestinal conditions^72^,^73^.

Another key signaling molecule, *TNFAIP3* (tumor necrosis factor, alpha-induced protein 3), a transcript also up-regulated both in gastrointestinal tissue and blood, is rapidly induced by TNF and inhibits NF-κB activation and TNF-mediated apoptosis. TNFα is present in both peripheral lymphocytes and inflamed small and large intestinal mucosal tissue in ASD patients, in excess of that found in TD^IC−^ and TD Crohn’s disease^17^,^18^,^74^. Moreover, variants of *TNFAIP3* are also known risk factors for celiac disease and are implicated in altered NF-κB signaling^75^.

The high affinity IgE receptor FcεR1 (consisting of one α subunit, one β subunit, and two γ subunits) is constitutively expressed in mast cells and basophils and initiates the allergic response upon interaction with allergens^76^. In humans, but not rodents, FcεR1 is also constitutively expressed in dendritic cells and monocytes, although this form of receptor is trimeric (lacking the β subunit) and it has been proposed that on dendritic cells, the receptor promotes immune homeostasis and regulation^77^. The gene encoding the alpha subunit for this receptor, *FCER1A*, is down-regulated in mucosal tissue and peripheral blood from ASD^IC+^ cases, which may further support the concept of an imbalance in immune homeostasis in ASD^IC+^.

We found that a key mitochondrial folate pathway gene, *MTHFD2* (methylenetetrahydrofolate dehydrogenase (NADP + dependent) 2, methenyltetrahydrofolate cyclohydrolase) encodes a nuclear-encoded bifunctional mitochondrial enzyme that is up-regulated in inflamed gastrointestinal tissue and down-regulated in peripheral blood in ASD^IC+^ cases. Importantly, this gene is expressed in developing embryos, but typically absent in most healthy adult tissues. *MTHFD2* RNA and protein are markedly elevated in many cancers and negatively correlated with survival in breast cancer^78^. Moreover, mitochondrial dysfunction is associated with ASD, although its role is unclear^79^,^80^.

Finally, *CYP2S1* (cytochrome P450, family 2), which encodes an extra-hepatic xenobiotic-metabolizing enzyme highly expressed in epithelial tissues (e.g. those in the lung, skin and colon^81^) is down-regulated in blood and in gastrointestinal tissue of ASD^IC+^ cases. CYP2S1 catalyzes reactions in drug metabolism and synthesis of cholesterol, steroids, and other lipids; some studies suggest it may play an important role in modulating inflammation. Whether it acts in an anti-inflammatory or pro-inflammatory mode depends on the substrate it encounters^82^. CYP2S1 is negatively regulated by corticosteroids, specifically dexamethasone, in human cell lines^83^. Glucocorticoids are widely used to treat allergic, inflammatory, and autoimmune conditions so this may provide one explanation for the reduced *CYP2S1* expression seen here.

Given that the current prevalence of ASD is 1 in 68 American children, the subset with gastrointestinal symptoms is substantial. An as yet unknown, but potentially high, fraction of these will have the ASD^IC+^ phenotype. Because empiric therapy for IBD will at best provide only short respite (and can even make symptoms worse), and because of the known association between severity of gastrointestinal symptoms and extremes of ASD behaviors, identifying children most likely to be ASD^IC+^ is critically important for clinicians to determine when diagnostic endoscopy is indicated.

We previously reported that clinical symptom presentation alone does not differentiate ASD children who do and do not have histologic evidence of chronic inflammatory bowel disease^9^. Children most likely to benefit from diagnostic gastrointestinal biopsy could therefore be reduced to manageable numbers by initial screening, using biomarkers, so that resources can be focused most appropriately.

Although the current study includes a more age- and gender-matched sample than our original pilot study^19^, the numbers of cases and controls are still relatively modest and constitute an important study limitation. Our findings merit validation in a much larger sample set and the existence of a putative nine-transcript biomarker will need to be replicated in blood samples of additional patients. ROC analyses suggest that a subset of the nine transcripts comprising the putative marker, consisting of three genes (*MTHFD2*, *IL1RN*, and *SIGLECP3*), provides a reasonable level of sensitivity and specificity with a combined AUC of 0.88.

Variations in age, gender, diet, medications, and nutritional supplements (used by many children in our cohort) may have impacted gene expression in the peripheral blood and gastrointestinal tract. However, controlling for these factors is nearly impossible in these cohorts.

We were able to match the sample groups for gender, but age matching is not feasible since gastrointestinal symptoms in ASD children typically present at a much earlier age than in their TD peers. This is one of the unique features of the ASD^IC+^ phenotype. Thus, gastrointestinal mucosa tissue based studies comparing cases and non-ASD controls would be expected to consist of groups with statistically significant differences in age and such studies have already appeared in the literature^84^.

Despite the potential confounds described above, our finding of gene expression profiles that consistently and convincingly segregate based on inflammation status in two different study cohorts effectively minimizes the potential effect of these confounding variables on the validity of the findings and speaks towards the authenticity of their presence as valid biomarkers.

There are additional limitations regarding the controls used in these studies. Although peripheral blood from ASD children with gastrointestinal symptoms can serve as a proxy tissue to indicate ileocolonic inflammation, our findings do not distinguish which of the nine unique blood-based DETs reflect the autism phenotype alone, the autism-plus-ileocolitis phenotype, or the ileocolitis phenotype alone. To address this limitation, our follow-on studies will include gene expression analysis in whole blood samples from two additional control groups: (1) ASD children without gastrointestinal symptoms and (2) typically developing children without gastrointestinal symptoms.

## Summary

In two separate case/control cohorts, we demonstrate overlap of 59 differentially expressed transcripts unique to inflamed ileocolonic tissues from ASD children with gastrointestinal symptoms. Nine of these 59 transcripts were also differentially expressed in the peripheral blood of ASD^IC+^ children from the second cohort. These nine transcripts could represent a putative blood-based biomarker for ASD-associated ileocolonic inflammation. Validation of these preliminary findings using two additional control cohorts (ASD without GI symptoms; TD without GI symptoms) and a larger ASD^IC+^ cohort are underway.

## Methods

### Case Selection and Biopsy Procurement

#### Participants

Our protocol was approved by the Wake Forest University Health Sciences Institutional Review Board (IRB approvals: #IRB00007834 [control samples] and #BG03-464 [ASD samples]) and informed consent was obtained from the parents of all study participants. All experiments were performed in accordance with relevant guidelines and regulations. Forty-eight sample sets from this IRB-approved study tissue bank were selected based on presence/absence of inflammation in the relevant tissue sample. For each subject, either 1 or 2 biopsies, and a single sample consisting of 2.5 ml whole blood collected into a PaxGene Blood RNA tube (PreAnalytiX), were processed.

The ASD^IC+^ group was selected based on a history of normal development for at least 12 months followed by developmental regression and onset of gastrointestinal symptoms. For all individuals in this group, this was their first diagnostic ileocolonoscopy; none was taking medication thought to alter histology of the gastrointestinal mucosa. All cases had histologically-confirmed ileitis, colitis, or both in at least one of seven collected and archived colonic biopsies.

Prior to being seen by the gastroenterologist, all patients had been assigned a diagnosis of either autism (N = 13), ASD (N = 4) or PDD-NOS (N = 4) (Table 1), given by one or more practitioners (pediatric neurologists, developmental pediatricians, pediatric psychiatrists, or psychologists). A detailed history of gastrointestinal symptoms was documented (Table 2). Patients who met clinical criteria for diagnostic ileocolonoscopy and biopsy and whose parents agreed to participate in this IRB-approved study (Copernicus Group Independent Review Board; WFU1-11-081) were provided with a study description and provided fully informed, written consent. Informed written consent from the next of kin, care givers or guardians on the behalf of all minor participants was obtained. Case specimens were obtained by one of the study authors (AK).

Specimens were obtained using a standard disposable forceps biopsy device, in accordance with routine diagnostic biopsy protocol. Immediately upon procurement of biopsy tissue, a specimen from each of seven anatomic locations (from the terminal ileum to rectum) was processed for paraffin embedding and subsequent routine histopathology. Biopsies for microarray analysis were obtained from the divided mucosal specimen at each anatomic location. These tissues were placed directly into RNAlater (Qiagen Inc.) and stored at −20 °C prior to processing.

#### Control biopsy procurement

Prospective controls (Table 1) were recruited through an IRB-approved protocol (Wake Forest University Health Sciences Institutional Review Board; #IRB00007834) from the Pediatric Gastroenterology Clinic at the Wake Forest University Health Sciences. The initial indication for ileocolonoscopy was presence of unexplained gastrointestinal symptoms (e.g. abdominal pain, diarrhea, malnutrition, blood observed in the stools). Control subjects were defined as those who, following ileocolonoscopy, had no endoscopic or pathologic findings to explain their symptoms. Failure to diagnose the etiology of observed symptoms by endoscopy was subsequently followed by clinical reassessment or additional diagnostic testing.

No concerns regarding developmental delays for any participant in the control group were reported by parents, relatives, caretakers, or teachers and none were noted by physicians at the Wake Forest Pediatric GI Clinic. Tissues for microarray analysis were collected, processed and stored in identical fashion to those from children with ASD. Informed written consent from the next of kin, care givers or guardians on the behalf of all the minors in all studies was obtained.

All specimens (cases and controls) were collected and stored in identical fashion (e.g. pinch cold biopsy forceps, immediate placement in RNAlater, and long-term storage at −20 °C within 24–48 hours post-collection). Cases were collected at two locations (Far Rockaway, NY and Austin, TX) with controls collected at a third location (Winston-Salem, NC).

#### Microarray Assay

RNA isolation from biopsy tissue samples was performed as described previously^19^. Briefly, mucosal biopsies stored in RNAlater were sonicated in the presence of TriReagent (Molecular Research Center, Inc., Cincinnati, OH) according to the method of Chomcynski and Sacchi^85^. Total RNA was purified using RNeasy Minelute Plus columns (includes an on-column DNAse step) and reagents (Qiagen, Valencia, CA) and eluted in nuclease-free water. RNA concentration and quality were determined using a Nanodrop ND-1000 (Nanodrop Technologies, Wilmington, DE) and Agilent Bioanalyzer, respectively. A single biopsy specimen was typically 3–5 mg of tissue and yielded 3 to 10 μg of high-quality (e.g. RIN ≥ 7) total RNA.

For the blood samples, total RNA was isolated in a QIAcube robotic workstation using RNeasyPlus kits (Qiagen) following the manufacturer’s protocols. RNA quantity and relative quality was assessed using a Nanodrop ND-1000 spectrophotometer. RNA integrity was determined using a bioanalyzer (Agilent Technologies, Palo Alto, CA). Total RNA for each sample (0.5–2.0 μg; RIN ≥ 7) was delivered to the Center for Genomics and Personalized Medicine Research Core Facility (Wake Forest Baptist Medical Center) for microarray assay, where labeled cDNA, generated from total RNA, was assayed on Illumina HT v4 BeadArray microarrays (Illumina Inc.). Following hybridization, washing, and scanning, data were extracted from scanned images using Genome Studio Software (Gene Expression module; Illumina Inc.) and processed for upload to gene expression analysis software.

#### TaqMan Validation

To provide further confirmation of a subset of microarray results, TaqMan PCR assays were employed. Briefly, all of the original peripheral blood RNA samples for which there was sufficient RNA remaining (19 of 21 cases and 20 of 24 controls) were used to generate cDNA (High-Capacity cDNA Reverse Transcription Kits; ABI) following the instructions provided with the reagents. Individual TaqMan PCR assays, representing 5 of the 9 transcripts listed in Table 3 (CYP2S1, TNFRSF12A, IL1RN, TNFAIP3 and SIGLECP3) were performed for each of the cDNAs, in triplicate wells on 96 well plates, in a StepOnePlus Real-Time PCR instrument (ABI). Delta C_T_ values were calculated by subtracting the average C_T_ for the reference gene (18S ribosomal RNA; also run in triplicate for each cDNA) from the average C_T_ of the sample for each of the five genes. Differential gene expression was calculated by comparing the relevant delta C_T_ for the cases and controls using the 2^−ΔΔCT^ method^86^ and the findings are reported in the Results section as a number representing “fold change” accompanied by an arrow indicating the direction of change.

#### Statistical Analysis

Raw data from the Illumina microarrays was imported into Genome Studio and, following quantile normalization (a process that transforms the raw data such that all arrays have a common distribution of intensities–similar to “scaling” in Affymetrix arrays) and log transformation, unsupervised hierarchical clustering, analysis of variance (ANOVA) and PCA were performed, using Qlucore Omics Explorer (Qlucore, Lund, Sweden), to generate principal component analysis (PCA) plots and heat maps. Individual comparisons between case and control groups were performed with Student’s t-test (fold change ≥1.5, p ≤ 0.05 for GI tissue; fc ≥ 1.2, p ≤ 0.05 for peripheral blood) using GeneSifter^®^ Analysis Edition (Perkin Elmer) software to generate lists of differentially-expressed genes (DEGs).

The p-values for the Illumina microarray data in this study are not corrected for multiple hypothesis testing (i.e. these are “raw p” versus “adjusted p” values) because in this second cohort study, due in part to the smaller number of cases and controls in each of the comparisons (12 vs 12 in the GI tissue comparisons), the false discovery rate correction eliminated a large number of the DETs that met criteria for FC ≥ 1.5 and p ≤ 0.05. For this reason, we chose to use the uncorrected data (i.e. raw p values) for comparison to the original dataset (wherein adjusted p values ≤ 0.05, following Benjamini Hochberg correction, were used as the cut-off) and then to perform qPCR validation in all peripheral blood samples from cohort #2 of a representative number of the overlapping nine genes.

Using only the genes that were differentially expressed in gastrointestinal tissue *and* peripheral blood, a receiver operating characteristic (ROC) curve analysis was also used to develop univariate and multivariable predictive models of blood-based biomarkers to discriminate ASD^IC+^ subjects from controls. The resulting multivariable model presents only those genes identified as significantly improving the model AUC based on a stepwise variable selection procedure.

All raw microarray data are available at GEO (Gene Expression Omnibus, Accession No. GSE87847, http://www.ncbi.nlm.nih.gov/geo/).

## Additional Information

**How to cite this article**: Walker, S. J. *et al.* A Putative Blood-Based Biomarker for Autism Spectrum Disorder-Associated Ileocolitis. *Sci. Rep.* **6**, 35820; doi: 10.1038/srep35820 (2016).

## Supplementary Material

Supplementary Information

Supplementary Table S2

Supplementary Table S4

Supplementary Table S5

Supplementary Table S6

Supplementary Table S3

## Figures and Tables

**Table 1:** Clinical and demographic characteristics of the study population.

	Cases ASD with inflammation	Controls TD without inflammation
Number	21	24
Age (years)		
Mean (SD)	8.11 (4.86)	12.66 (4.08)
Range	2.3–19.25	2.08–17.9
Gender		
Male (%)	17 (81)	18 (75)
Female (%)	4 (19)	6 (25)
Diagnosis		
Autism	13	—
ASD	4	—
PDD-NOS	4	—

**Table 2:** Gastrointestinal symptoms in the ASD study population.

	ASD^IC+^ N = 21 n (%)	Non-ASD^IC−^ N = 24 n (%)
Abdominal pain	18 (86)	12 (50)
Diarrhea	16 (76)	7 (29)
Constipation	8 (38)	1 (4)
Food sensitivities	8 (38)	0 (0)
Abdominal distention	7 (33)	0 (0)
Failure to thrive	5 (24)	5 (21)

**Table 3:** Student’s t-test was used to measure differential expression (@ fc ≥ 1.2; p ≤ 0.05) of nine transcripts in comparison of tissues from two independent studies.

Accession #	Gene Symbol	Study #1 ASD-TI	ASD-colon	Study #2 ASD-TI	ASD-colon	blood
NM_002001	FCER1A	2.69 ↓ (0.003)	3.52 ↓ (2.75E-07)	3.11 ↓ (0.0003)	1.94 ↓ (0.003)	1.55 ↓ (0.037)
NM_030622	CYP2S1	2.56 ↓ (9.48E-06)	2.76 ↓ (4.76E-09)	2.31 ↓ (0.003)	1.63 ↓ (0.007)	2.47 ↓ (0.035)
NM_144686	TMC4	2.36 ↓ (3.13E-06))	1.59 ↓ (0.0005)	2.11 ↓ (0.0015)	1.55 ↓ (0.0001)	3.16 ↑ (0.036)
NM_016639	TNFRSF12A	1.96 ↓ (0.0004)	3.16 ↑ (0.0008)	1.75 ↓ (0.048)	2.98 ↑ (0.028)	2.95 ↓ (0.033)
NM_173842	IL1RN	1.94 ↑ (0.0009)	3.03 ↑ (0.003)	6.91 ↑ (0.001)	4.22 ↑ (0.009)	1.41 ↓ (0.009)
NM_006290	TNFAIP3	1.75 ↑ (0.0002)	1.62 ↑ (0.006)	1.67 ↑ (0.016)	1.53 ↑ (0.02)	1.26 ↑ (0.018)
NM_001813	CENPE	1.66 ↑ (0.015)	2.71 ↑ (2.61E-07)	1.77 ↑ (0.0017)	1.51 ↑ (0.0006)	3.46 ↓ (0.01)
NM_006636	MTHFD2	1.66 ↑ (0.003)	2.25 ↑ (2.57E-05)	1.85 ↑ (0.0027)	1.83 ↑ (0.0004)	1.78 ↓ (0.03)
NR_002804	SIGLECP3	1.63 ↓ (0.006)	1.81 ↓ (0.004)	3.69 ↓ (0.023)	4.83 ↓ (0.002)	3.64 ↓ (0.008)

The direction of change is indicated by the arrows and p value is indicated in parentheses beneath the fold change.
