# Supplementary material for: A Putative Blood-Based Biomarker for Autism Spectrum Disorder-Associated Ileocolitis
Source: Sci Rep. 2016 Oct 21;6:35820. doi: 10.1038/srep35820 (PMC5073317; doi:10.1038/srep35820)
Supplement: Supplementary Information [file srep35820-s1.pdf]

A Putative Blood-Based Biomarker for Autism Spectrum Disorder-Associated Ileocolitis

Stephen J. Walker[1,*], Daniel P. Beavers[2], John Fortunato[3] & Arthur Krigsman[4]

[1]Wake Forest Institute for Regenerative Medicine, Wake Forest University Health Sciences, Winston Salem, NC, [2]Department of Biostatistical Sciences, Public Health Sciences, Wake Forest University Health Sciences, Winston Salem, NC, [3]Pediatric Gastroenterology, Hepatology, and Nutrition, Ann & Robert H. Lurie Children's Hospital of Chicago, Chicago, IL, [4]Pediatric Gastroenterology Resources, 148 Beach 9th Street, Suite 2B,Far Rockaway, NY

*swalker@wakehealth.edu

Supporting Information

**Table S1**	**59 transcripts differentially regulated in both terminal ileum and colon in ASD^{IC+} samples from this study and our earlier pilot study.**
**Table S2**	**3183 transcripts differentially regulated in peripheral blood in ASD^{IC+} samples compared to TD controls from the 2nd cohort study.**
**Table S3**	**2949 transcripts differentially regulated in colon in ASD^{IC+} compared to TD control samples from our earlier pilot study.**
**Table S4**	**2821 transcripts differentially regulated in colon in ASD^{IC+} compared to TD control samples from this 2nd cohort study.**
**Table S5**	**3497 transcripts differentially regulated in terminal ileum in ASD^{IC+} compared to TD control samples from our earlier pilot study.**
**Table S6**	**2451 transcripts differentially regulated in terminal ileum in ASD^{IC+} compared to TD control samples from this 2nd cohort study.**

Figure S1 **Univariate ROC curves for the nine transcripts differentially expressed in mucosal tissue and blood in ASD^{IC+} individuals.**

Please note:

Table S1 and Figure S1 are provided in this document. Due to their size, and to preserve formatting, Tables S2 – S6 are provided as individual excel spreadsheets.

Table S1 - Transcripts that are differentially expressed in inflamed ileum and colon in ASDIC+ cases from two studies

Gene Name	Gene Identifier
neurotensin	NM_006183
V-set and immunoglobulin domain containing 2	NM_014312
neuropeptide Y	NM_000905
gamma-aminobutyric acid type A receptor alpha2 subunit	NM_000807
aquaporin 11	NM_173039
testis-specific transcript, Y-linked 15 (non-protein coding)	NR_001545
WAP four-disulfide core domain 1	NM_021197
ephrin-A1	NM_004428
interleukin 2 receptor subunit alpha	NM_000417
Fc fragment of IgE receptor Ia	NM_002001
solute carrier family 22 member 4	NM_003059
cytochrome P450 family 2 subfamily S member 1	NM_030622
meprin A subunit alpha	NM_005588
N-acetylated alpha-linked acidic dipeptidase-like 1	NM_005468
RAB17, member RAS oncogene family	NM_022449
biotinidase	NM_000060
solute carrier family 26 (anion exchanger), member 2	NM_000112
vasoactive intestinal peptide receptor 1	NM_004624
transmembrane channel like 4	NM_144686
meprin A subunit beta	NM_005925
glucosidase, beta, acid 3 (gene/pseudogene)	NM_020973
period circadian clock 3	NM_016831
troponin C2, fast skeletal type	NM_003279
ornithine carbamoyltransferase	NM_000531
glutathione S-transferase mu 2 (muscle) pseudogene 1	NR_002932
actin binding LIM protein family member 2	NM_032432
thyrotrophic embryonic factor	NM_003216
formyl peptide receptor 1	NM_002029
epoxide hydrolase 1	NM_000120
tumor necrosis factor receptor superfamily member 12A	NM_016639
interleukin 1 receptor antagonist	NM_173842
ribosomal protein L39 like	NM_052969
LDL receptor related protein 8	NM_033300
cyclic nucleotide gated channel alpha 1	NM_000087
Cdk5 and Abl enzyme substrate 1	NM_138375
membrane spanning 4-domains A10	NM_206893
high mobility group box 2	NM_002129

centromere protein L	NM_033319
dishevelled-binding antagonist of beta-catenin 2	NM_214462
TNF alpha induced protein 3	NM_006290
zinc finger protein 358	NM_018083
polo like kinase 4	NM_014264
shugoshin-like 2 (S. pombe)	NM_152524
TIMELESS interacting protein	NM_017858
kinesin family member 14	NM_014875
solute carrier family 27 member 2	NM_003645
centromere protein E	NM_001813
methylenetetrahydrofolate dehydrogenase 2, methenyltetrahydrofolate cyclohydrolase	NM_006636
ubiquitin like with PHD and ring finger domains 1	NM_013282
SPC25, NDC80 kinetochore complex component	NM_020675
sulfiredoxin 1	NM_080725
sialic acid binding Ig like lectin 17, pseudogene	NR_002804
replication factor C subunit 3	NM_002915
BUB1 mitotic checkpoint serine/threonine kinase B	NM_001211
helicase, lymphoid-specific	NM_018063
non-SMC condensin I complex subunit G	NM_022346
cytochrome P450 family 51 subfamily A member 1	NM_000786
calcium voltage-gated channel auxiliary subunit alpha2delta 4	NM_172364
paired like homeodomain 1	NM_002653

Figure S1 **Univariate ROC curves for the nine transcripts found to be differentially expressed in mucosal tissue and blood in ASD^{IC+} individuals.**

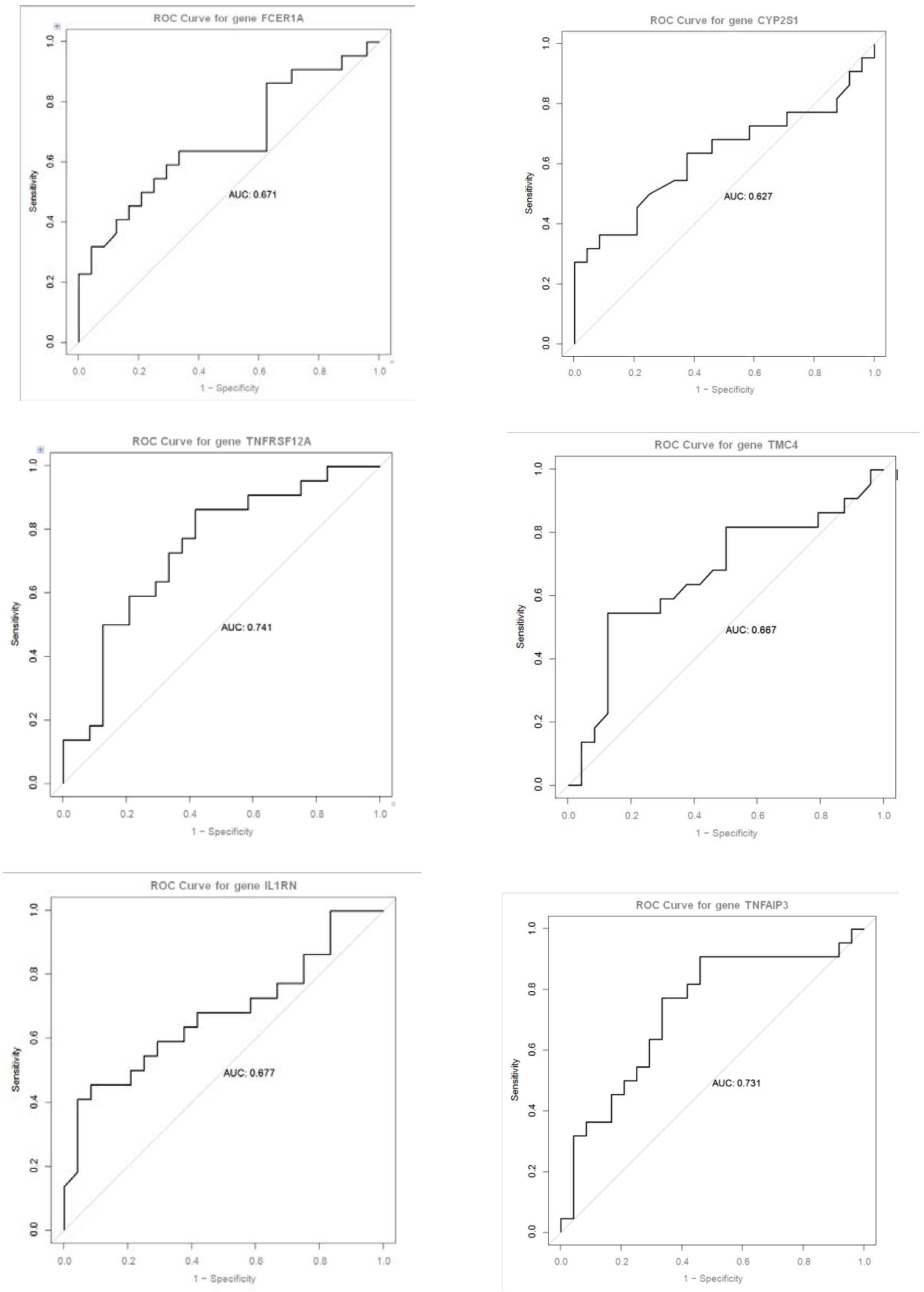

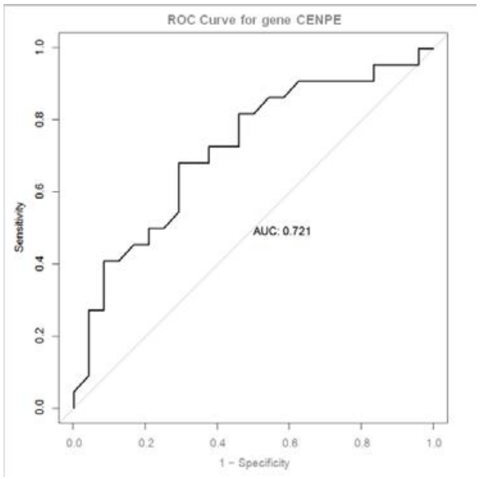
ROC Curve for gene CENPE
Sensitivity
1 - Specificity
AUC: 0.721
0.0
0.2
0.4
0.6
0.8
1.0

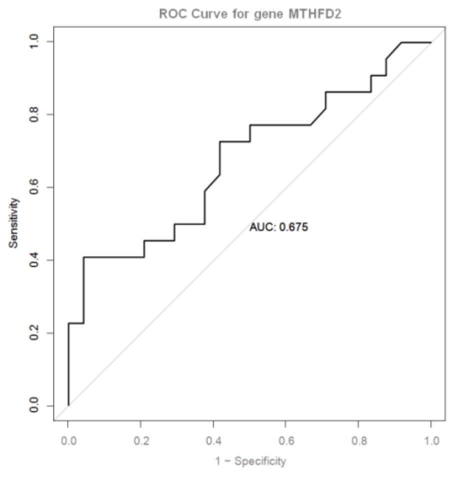
ROC Curve for gene MTHFD2
Sensitivity
1 - Specificity
AUC: 0.675
0.0
0.2
0.4
0.6
0.8
1.0

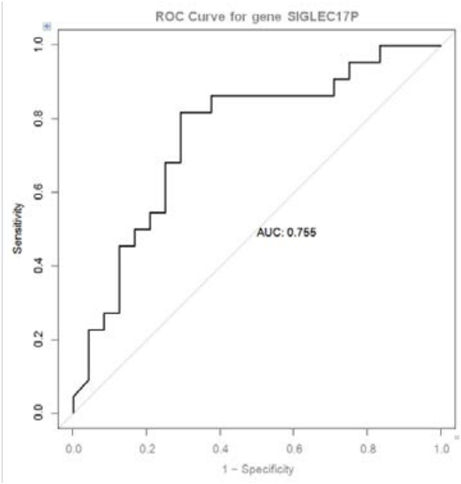
ROC Curve for gene SIGLEC17P
Sensitivity
1 - Specificity
AUC: 0.755
0.0
0.2
0.4
0.6
0.8
1.0
